# Application of Ohmic–Vacuum Combination Heating for the Processing of Senior-Friendly Food (Multiphase Food): Experimental Studies and Numerical Simulation

**DOI:** 10.3390/foods10010138

**Published:** 2021-01-11

**Authors:** Sung Yong Joe, Jun Hwi So, Seon Ho Hwang, Byoung-Kwan Cho, Wang-Hee Lee, Taiyoung Kang, Seung Hyun Lee

**Affiliations:** 1 Department of Biosystems Machinery Engineering, Chungnam National University, Daejeon 34134, Korea; dhrmaksdyd@naver.com (S.Y.J.); chobk@cnu.ac.kr (B.-K.C.); wanghee@cnu.ac.kr (W.-H.L.); 2 Department of Smart Agriculture Systems, Chungnam National University, Daejeon 34134, Korea; sjha24@naver.com (J.H.S.); hbs3689@naver.com (S.H.H.); 3 Department of Molecular Biosciences and Bioengineering, University of Hawaii, Honolulu, HI 96822, USA

**Keywords:** senior-friendly food, solid–liquid mixture, ohmic heating, vacuum

## Abstract

The popularity of senior-friendly food has been increasing as the world enters the age of an aging society. It is required that senior-friendly food products are processed with the new concept of processing techniques that do not destroy the nutritional and sensory values. Ohmic heating can be an alternative to conventional heating methods for processing senior-friendly food with retaining excellent taste and quality because of less destruction of nutrients in the food. In this study, the ohmic–vacuum combination heating system was developed to process a multiphase type of senior-friendly food. Changes in physical and electrical properties of senior-friendly model foods were investigated depending on the experimental conditions such as vacuum pressure intensity and vacuum pretreatment time. Numerical simulations based on the experimental conditions were performed using COMSOL multiphysics. The ohmic–vacuum combination heating method with agitation reduced the heating time of the model food, and non-uniform temperature distribution in model food was successfully resolved due to the effect of vacuum and agitation. Furthermore, the difference was found in the hardness of solid particles depending on the vacuum treatment time and intensity after the heating treatment. The ohmic–vacuum combination heating system appeared effective when applying for the senior-friendly foods in multiphase form. The simulation results matched reasonably well with the experimental data, and the data predicted through simulation could save the cost and time of experimentation.

## 1. Introduction

The world’s older population has risen over the past few years. Especially, the number of elderly people in Korea and Japan is set to grow at an unprecedented rate because the fertility rate has been drastically decreased [1,2,3]. According to the portion of the older population, it can be classified into aging society (7% or more), aged society (14% or more), and super-aged society (20% or more) [4]. Japan already entered the super-aged society in 2006, and the older population is expected to exceed 40% of the total population by 2050 [5,6]. Korea is also becoming an aging society. Since a large portion of the elderly population suffers from eating disorders such as masticatory disorders, swallowing disorders, and digestive disorders, they cannot take the essential nutrition from foods [7]. Eating disorders can inevitably lead to an unbalanced diet, which causes malnutrition in the elderly population [8,9]. Malnutrition leads to a decrease in muscle and blood volume, especially causing a rapid decrease in physical function [10,11]. An overall lack of energy can also lead to a vicious cycle of decreased appetite as vitality and activity decrease [12]. To overcome the nutrient intake problem of the elderly people, the interest in senior-friendly foods that can reflect the physical characteristics of elderly consumers and satisfy various tastes is growing [13]. Senior-friendly foods refer to all kinds of food manufactured and processed for the purpose of substituting general meals or keeping the body healthy for the elderly people suffering from metabolic functions. Senior-friendly foods, which have a strong characteristic of patient food, have been developed to satisfy the demand from elderly consumers with an emphasis on maintaining taste and shape. Therefore, these foods should focus on retaining food properties and nutrients during processing so that elderly consumers can easily take enough nutrients.

Conventional cooking methods require long processing time and have difficulty maintaining food quality such as flavor, aroma, texture and appearance [14,15]. In order to process senior-friendly foods, it is necessary to develop new thermal processing technology that enables proper sterilization (or pasteurization) while preserving nutrients in the food through minimum thermal treatment [16]. Among food thermal treatment methods, ohmic heating (OH) has been widely used for sterilization and pasteurization of heat sensitive food products. Resistance heat can be generated inside the food product during OH by passing alternative current through the food product with close contact to electrodes. Compared with other thermal treatment methods (i.e., microwave heating, infrared heating), the OH of single-phase food (liquid or solid phase food) can provide thermal uniformity enhancement, high heating rate and energy conversion efficiency [17,18]. For high-temperature short-time (HTST) sterilization of multiphase foods containing solid particles, OH allows large solid particles to be simultaneously treated with liquid phase food, which was not possible using conventional heat exchangers [19]. However, the OH heating rate significantly depends on electrical conductivities of foods. Since non-uniform heating of multiphase during OH can occur due to the differences of electrical conductivities between the solid phase and liquid phase [20], it can lead to over or under-treatment of multiphase food, consequently resulting in quality deterioration.

The ohmic–vacuum (OH–VC) combination heating method has not been well investigated [21]. During the OH of liquid phase food, the temperature of liquid continues to rise until it reaches its boiling point [22]. To keep its temperature constant, the power source must be controlled or some cooling medium must be applied during the heating process [23]. However, by combining OH with vacuum, the boiling temperature of liquid can be lowered [24]. When the liquid food reaches the boiling point, its temperature remains constant as long as the vacuum pressure does not change [25]. The uniform temperature distribution of multiphase food during OH–VC combination heating can be obtained by controlling the boiling point of liquid phase food using a vacuum [16]. A vacuum combination heating method can improve energy efficiency and the texture of the processed product [26]. Controlling the exact temperature distribution of multiphase food plays an important role in the OH process; however, it is complicated because of the heat transfer between solid and liquid phases [27,28]. In order to determine the temperature distribution of multiphase food in OH, a number of parameters should be experimentally evaluated and complex numerical methods should be applied [29,30]. Magnetic resonance imaging (MRI) temperature mapping was used to observe the temperature distribution of multiphase food during OH [29,31]. Even though temperature distribution could be observed in real time, the cost of MRI was relatively high and the additional space was required [27]. The use of computational simulation can accurately predict temperature distribution of multiphase food under OH [32]. Understanding the behavior of the OH process is essential to demonstrate the correct reliability of the heating system and to the safety of the process [33]. Numerical modeling provides an insight into the heating behavior of OH [34]. Temporal and spatial temperature distributions of multiphase food during OH can be provided from the reliable numerical model [35]. 

This study was conducted to develop an OH–VC combination heating system for processing senior-friendly food products consisting of solid particles and liquid, and to determine temperature uniformity of multiphase foods under OH–VC combination heating depending on the presence of agitation. In addition, computational fluid dynamic (CFD) models were developed to validate the multiphase food OH process with agitation. As far as can be determined from the accessible literature, the CFD model for OH of multiphase food with agitation was not attempted and developed. 

## 2. Materials and Methods 

### 2.1. Preparation of Model Food

The solution model food (base solution) used in this study was prepared by imitating commercialized senior-friendly foods (2 types of soft diets (A, B), 3 types of liquid foods (C, D, E), and 1 pudding food (F)). The constituents, viscosity, and electrical conductivity of six different types of commercialized senior-friendly foods purchased from the local silver food market were analyzed prior to preparing the base solution. The samples were stored in 4 °C refrigeration condition. Samples were taken out at room temperature for 1 h before the experiment, and the viscosity and electrical conductivity of the samples were measured at room temperature (around 21 to 23 °C). The viscosity of the samples was measured by using a viscometer (CL-L2, CAS, Inc., Seoul, Korea). 

The electrical conductivities of the samples were determined using a custom-made Teflon OH test cell (0.01 m in inner diameter and 0.1 m in height) consisting of food grade stainless steel (SUS 316) electrodes (0.01 m in diameter) that were placed at both ends of OH cell through a pair of spacers. During the electrical conductivity measurement, the sample temperature was measured using a thermocouple (K-type KK-K-30, Omega Engineering Inc., Stamford, CT, USA), which was inserted at the center of the sample through a small hole on the surface of the OH test cell. The electrical conductivities of samples were calculated by the following equation;

(1) $$\sigma =\frac{I}{V}\cdot \frac{L}{A}$$

where σ is the electrical conductivity (S/m), *I* is the current (A), *V* is the applied voltage (V), *L* is the distance between the electrodes (m), and *A* is the contact area between electrode and sample (m^2^). 

The viscosity of the samples ranged from 20 to 160 cPs above 80% torque. The measured electrical conductivities of the samples as a function of temperature are summarized in Table 1. Based on the measured viscosity and electrical conductivity of samples, the base solution was made by using whole milk powder (Seoul milk, Seoul, Korea) and black bean soup (Chung’s Food Co., Ltd., Cheongju, Korea). The mixing ratio of the base solution was 1 (whole milk powder) to 6 (black bean soup). The viscosity of the base solution was 100 cPs at over 80% torque, and the electrical conductivity of the base solution was linearly increased with increasing temperature (Table 1). 

Pork sirloin purchased from a local butcher shop was used as solid particles, and were cut into cubes (2 × 2 × 2 cm). The solid cubes were added into the base solution to produce model senior-friendly food (mixture food). The solid fraction in the total volume of mixture food was 10/100 g. The amount of model food for each experiment was 800 g.

### 2.2. Ohmic–Vacuum Combination Heating System 

**Figure 1:**
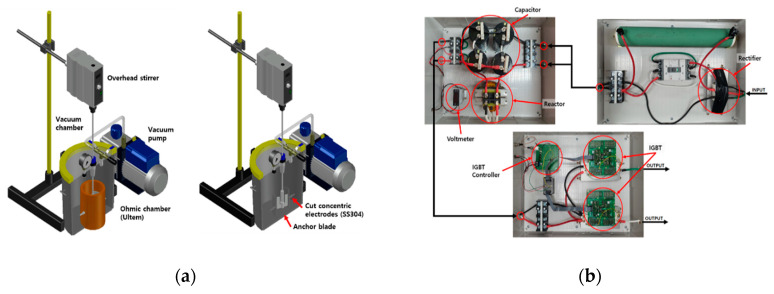
Ohmic–vacuum combination heating system: (**a**) schematic diagram of system, (**b**) ohmic heating power supply configuration.

The ohmic–vacuum combination heating system is shown in Figure 1. The system consisted of a vacuum chamber and pump, ohmic chamber, a pair of curved rectangular electrodes, overhead stirrer, and ohmic power supply. The vacuum chamber could maintain a vacuum gauge pressure of up to 0.1 bar. The cylindrical ohmic chamber (outer diameter of 12.7 cm, inner diameter of 10.1 cm, and height of 18 cm) was made of Ultem (PEI) to prevent distortion of the chamber by vacuum pressure and heat. Curved rectangular electrodes with circumference of 9.8 cm, thickness of 0.2 cm, and height of 10 cm were fabricated by cutting sanitary stainless steel (SUS 304 pipe), and installed parallel to both sides of the chamber. Ohmic power supply was designed and custom built by using the IGBT module (404GB12E4s, SKYPER 42R driver and board, Semikron, Inc., Hudson, NH, USA). The power supply was able to generate pulsed alternating current with a frequency range between 1 Hz and 20 kHz, on/off duty cycle from 0.2 to 0.8, and maximum current of 30 A at 380 V_rms_. 

The ohmic chamber was placed at the bottom center of the vacuum chamber. To measure temperature during ohmic–vacuum combination heating, 2 k-type thermocouples were installed at the middle and bottom part of the ohmic chamber through a small hole of the vacuum chamber lid. The curved rectangular electrodes and ohmic power supply were connected similarly to thermocouple installation. An anchor blade-stirring bar was inserted at the center of the ohmic chamber through a vacuum stirring seal installed at the center of the vacuum chamber lid. The stirring bar was connected with an overhead stirrer (ms5020D, Misung Scientific, Seoul, Korea). The vacuum stirring seal was effective in preventing air inflow during ohmic–vacuum combination heating. All data points of temperature, voltage, and current were monitored and recorded by using a differential probe (PR-60, BK Precision, Yorba Linda, CA, USA), wideband current monitor (169820, Pearson Electronics, Palo Alto, CA, USA), oscilloscope (DPO 4034, Tektronix, Beaverton, OR, USA), and data acquisition unit (DAQ) (39704A, Agilent, Palo Alto, CA, USA). 

### 2.3. Measurement of Hardness of Solid Particles

**Figure 2:**
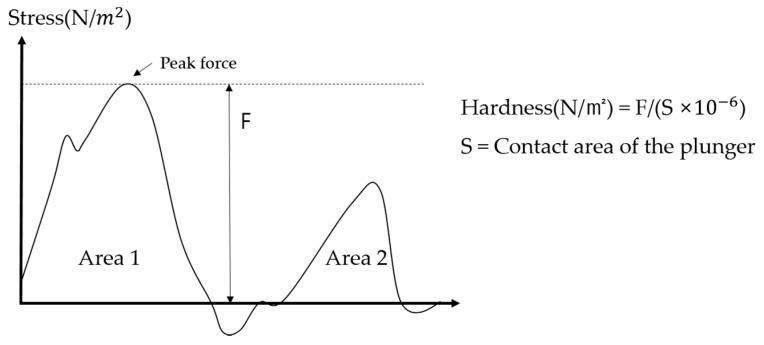
Diagram of Texture Profile Analysis (TPA) hardness measurement.

Texture Profile Analysis (TPA) was conducted using a texture analyzer (TA.XT Plus, Texture Technologies, Scarsdale, NY, USA) to evaluate the change in hardness of solid particles in the model food after OH–VC combination heating depending on vacuum pressure and vacuum pretreatment time. TPA mimics the chewing effect of putting food in the mouth and chewing it with teeth. It is a method of analyzing the physical properties of food by applying compression force twice consecutively (Figure 2). The hardness of solid particles was measured using a 30 mm diameter circular probe. Solid particles and liquid particles were immediately separated after the OH experiment, and then the hardness of solid particles was analyzed. 

### 2.4. Experimental Design

The boiling point of the base solution during OH was investigated depending on the change in vacuum pressure strength and the presence of agitation. The vacuum pressure was controlled within the range of 1.01325 bar (1 atm) to 0.1 bar. The OH experiment was immediately stopped when the liquid boiled at different vacuum intensity levels by applying a voltage of 70 V, duty cycle of 50%, and frequency of 15 kHz. Thermocouples were installed in the middle and bottom of the heating chamber to monitor temperature values of the base solution.

The temperature uniformity of the model food containing pork cubes and base solution during OH was evaluated depending on the presence of agitation. In addition, the effect of vacuum pretreatment on change in temperature of the model food was investigated. The model food was pretreated for 5, 10, and 10 min at different vacuum intensity levels (vacuum gauge pressure: 0.8, 0.5, 0.2 bar, and atmospheric pressure) under agitation before applying voltage to the heat model food. Then, 100 V at frequency of 15 kHz with duty cycle of 50% was applied for the OH of the model food, while maintaining the same vacuum pressure intensity as the vacuum pretreatment condition. In this study, this heating method was named as the ohmic and vacuum (“OH–VC”) combination heating with agitation. The temperature of the base solution was measured through the thermocouples (K-type KK-K-30, Omega Engineering Inc., Stamford, CT, USA) installed in the middle and bottom of the heating chamber. The temperature values of pork particles were measured by inserting thermocouples to the core of the particles as soon as the heating process was finished. The OH–VC combination heating experiment of the model food was stopped when the middle or bottom temperature of the base solution reached 90 °C. As soon as OH experiments were completed, solid particles and base solution were immediately separated from the model food to evaluate the effect of vacuum pretreatment conditions on change in hardness of solid particles. 

**Figure 3:**
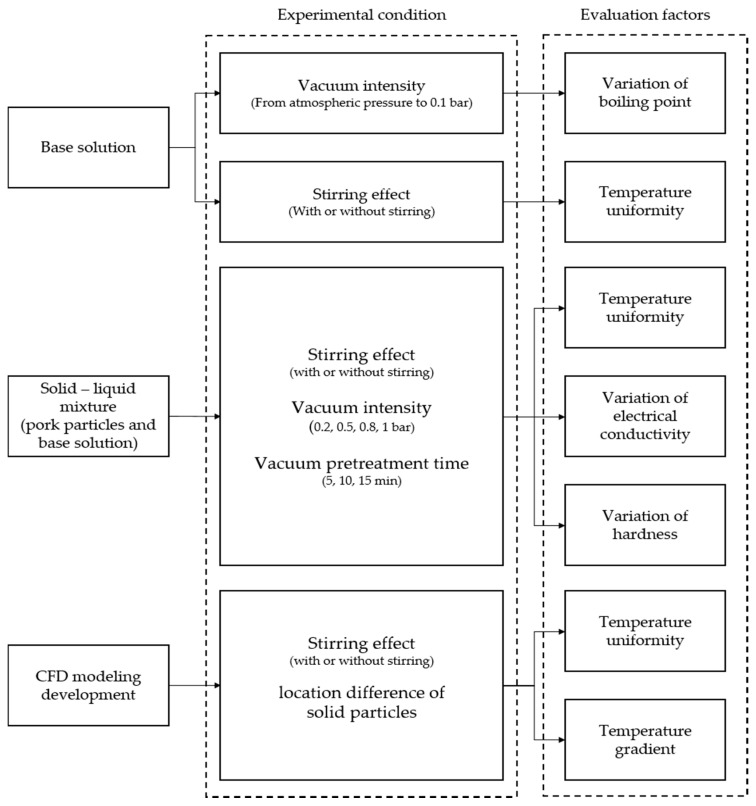
A schematic of overall experimental protocol.

Furthermore, the effect of vacuum pretreatment on change in the electrical conductivities of the model food was investigated. After the model food was pretreated by different vacuum pressure intensity levels for different times, solid particles and base solutions were separated and then both electrical conductivities were measured by using the aforementioned method in Section 2.1. The overall experimental protocol is illustrated in Figure 3.

### 2.5. Mathematical Modeling

The mathematical modeling was developed to understand and predict the heat transfer and heat distribution in multiphase food during ohmic heating combined with agitation by using COMSOL Multiphysics software (COMSOL 5.5, COMSOL, Inc., Palo Alto, CA, USA) including AC/DC, Heat Transfer, and CFD modules. 

#### 2.5.1. Governing Equation for Electromagnetic Heat Generation

The electric field distribution in the ohmic heater was determined by using the Laplace equation [18];

(2) ∇·(σ∇V)=0

where V is the voltage (V), ∇ is the gradient, σ is electrical conductivity (S/m). 

Since electrical conductivities of foods are a function of temperature, temperature and electrical conductivity can be expressed in a linear relationship [19];

(3) $$\sigma_{i}\left(T\right)=\;\sigma_{0}\left(1+mT\right)$$

where $\sigma_{0}$ is the reference value (S/m), m is the temperature coefficient, T is the temperature (K). 

The heat source of the ohmic heating simulation was heat generation inside the food by electric current. Internal energy generation during the OH of multiphase food by conduction can be determined by the following equation [31];

(4) $$\rho C_{p}\frac{\partial T}{\partial t}=\nabla k\nabla T+Q_{gen}$$

where ρ is the sample density (kg/m^3^), *C_p_* is the specific heat (kJ/kgK), *T* is the temperature within the sample (K), *t* is the heating time (s), *k* is the thermal conductivity (W/m °C). 

The heat generation (*Q_gen_*) during ohmic heating is proportional to the electrical conductivity of food and the square of the voltage gradient [18];

(5) $$Q_{gen}=\;\sigma {\left|\nabla V\right|}^{2}$$

#### 2.5.2. Governing Equation for Turbulent Flow

The flow of the model food during OH was caused by agitation. The flow by agitation complicates the determination of the temperature distribution in multiphase food. Therefore, turbulent flow analysis was added to identify the exact temperature distribution inside the food. The flow of model food during OH was determined by the k−ε turbulence equation and turbulent velocity ($u_{T}$) is significantly affected by turbulent kinematic energy (*k*) and the turbulent dissipation rate (ε) [36];

(6) $$u_{T}=\rho C_{\mu }\frac{k^{2}}{\varepsilon }$$

where $u_{T}$ is the turbulent velocity (m/s), ρ is the density (kg/m^3^), $C_{\mu }$ is the constant model parameter, *k* is the turbulent kinetic energy (m^2^/s^2^), and ε is the turbulent dissipation rate (m^2^/s^3^). 

Turbulent kinetic energy (*k*) can be calculated by following the transportation equation [36];

(7) $$\rho \frac{\partial k}{\partial t}+\rho u\cdot \nabla k=\nabla \left[\left(\mu +\frac{\mu_{T}}{\sigma_{k}}\right)\nabla k\right]+P_{k}-\rho \varepsilon$$

(8) $$P_{k}=\mu_{T}\left[\nabla u:\left(\nabla u+{\left(\nabla u\right)}^{T}\right)-\frac{2}{3}{\left(\nabla \cdot u\right)}^{2}\right]-\frac{2}{3}\rho k\nabla \cdot u$$

where u is the mean velocity (m/s), μ is the dynamic viscosity (Pa·s), $\mu_{k}$ and $\sigma_{k}$ are the constant model parameters, and $P_{k}$ is the production term.

An additional transportation equation is necessary for the calculation of and turbulent dissipation rate (ε) [36];

(9) $$\rho \frac{\partial \varepsilon }{\partial t}+\rho u\cdot \nabla \varepsilon =\nabla \cdot \left[\left(\mu +\frac{\mu_{T}}{\sigma_{\varepsilon }}\right)\nabla \varepsilon \right]+C_{\varepsilon 1}\frac{\varepsilon }{k}P_{k}-C_{\varepsilon 2}\rho \frac{\varepsilon^{2}}{k}$$

where $C_{\varepsilon 1}$ and $C_{\varepsilon 2}$ are the constant model parameters. 

#### 2.5.3. Mathematical Modeling Setup

Mathematical modeling proceeded in the following order: (1) creation of geometry for modeling, (2) initial and boundary condition assignment, (3) mesh generation and optimization, (4) solver selection, (5) tolerance and time step setting, and (6) built-in convergence solution.

The boundary conditions of the heat transfer equation assumed that all samples were thermally insulated, and the initial temperature values of the entire samples (solid particles and base solution) were set to 303.15 K. The thermal properties of model food were calculated based on experimental data. The specific heat, density, and thermal conductivity of the base solution and solid (pork) particles were 3.9 and 2.71 kJ/kg∙K, 1030 and 1099.7 kg/m^3^, and 0.5948 and 0.21 W/m∙K, respectively. The electrical conductivity was increased with an increase in temperature. The electrical conductivities of the base solution and solid particles as a function of temperature were set to 0.01035×(T−273.15) + 0.61915 S/m and 0.0171×(T−273.15) + 0.5813 S/m, respectively. In order to improve the mesh quality, the computational domain was discretized with tetra meshes. The mesh geometry consisted of 11,515 tetrahedrons, 1328 boundary elements, and 392 edge elements. The direct linear system solver (PARDISO) was used to increase the convergence rate. The relative tolerance and absolute tolerance used in PARDISO was 0.05. 

**Figure 4:**
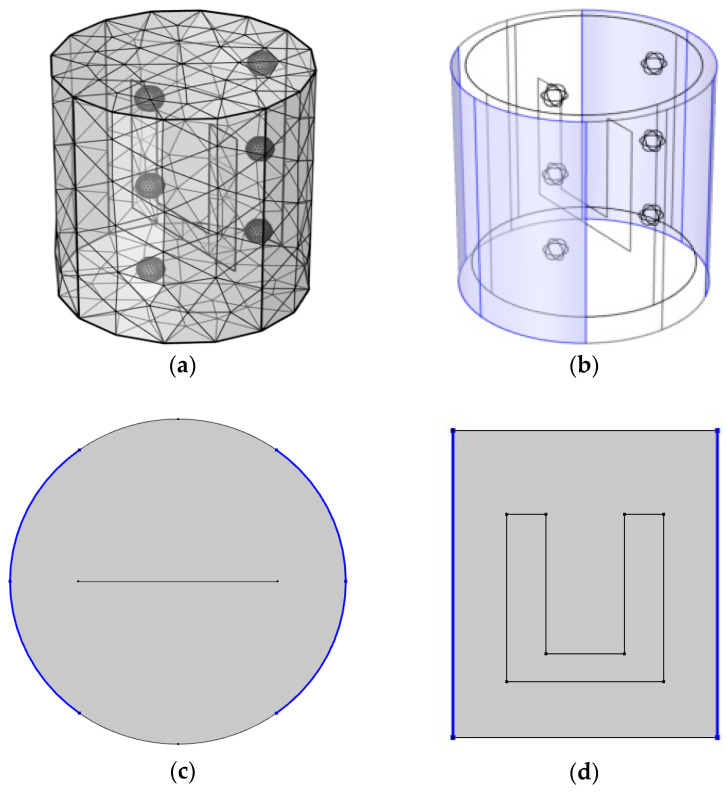
Geometry used in the ohmic heating simulation: (**a**) grid mesh geometry, (**b**) electrode layout, (**c**) top view, and (**d**) front view.

The geometry used in the simulation is shown in Figure 4. A pair of electrodes were placed on both sides of a cylinder with a diameter of 101 mm and a height of 110 mm. The shaded parts in blue (Figure 4b) are the electrodes and the applied voltage for simulation was 100 V. A stir head shape was employed to mimic the stirring activity and the applied rotation speed was 60 rpm. A spherical particle with a diameter of 10 mm was added to evaluate the temperature difference between solid and liquid. The particles were located at six points to determine the temperature difference depending on the location.

## 3. Results and Discussion

### 3.1. The Effect of Vacuum Pretreatment on Change in Electrical Conductivities of Pork Particle and Base Solution

After pork particles and the base solution were pretreated at different vacuum pressure intensity levels for different times under agitation, their electrical conductivities were measured (Table 2). The electrical conductivities of solid particles and base solution without vacuum pretreatment were compared to those with vacuum treatment. Regardless of vacuum pretreatment, the electrical conductivities of both samples were linearly increased with an increase in temperature. The vacuum pretreatment caused a slight increase in the electrical conductivities of both samples. The electrical conductivities tended to decrease with increasing vacuum pretreatment time; however, the difference resulting from pretreatment times was not significant. In vacuum pretreatment processing of multiphase food, the enhanced osmotic pressure by vacuum pretreatment caused the change in the electrical conductivity of solid particles by increasing the solute or water absorption of solid particles from the solution [37]. The vacuum pretreatment used in this study led to the change in the electrical conductivities of both samples by affecting the mutual movement of electrolytes or moisture between the solid and liquid.

### 3.2. Change of Boiling Point of Base Solution Depending on Vacuum Intensity

**Figure 5:**
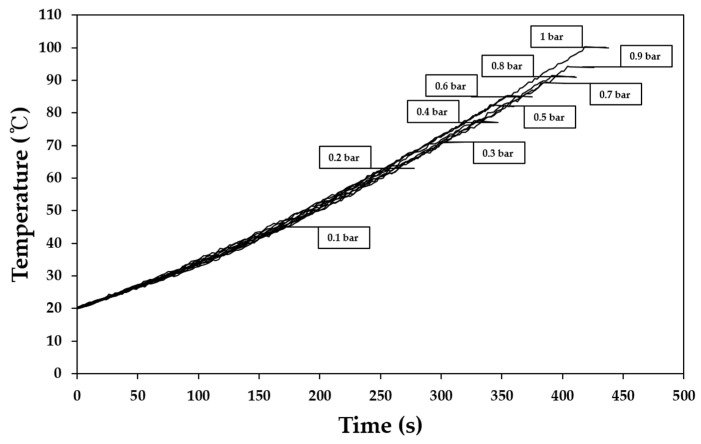
Change of boiling point of base solution under different vacuum intensities.

The base solution boiled at 97 °C under atmospheric pressure, and the boiling point decreased by approximately 3 °C as the vacuum gauge pressure decreased until 0.4 bar at a decrement interval of 0.1 bar, as shown in Figure 5. The boiling point rapidly decreased below 0.3 bar, and the boiling point at 0.1 bar was 45 °C, which was more than 50 °C below the boiling point under atmospheric pressure. It was clearly observed that the boiling point was significantly dependent on vacuum intensity. 

### 3.3. The Effect of Agitation on Temperature Uniformity of Base Solution and Model Food

**Figure 6:**
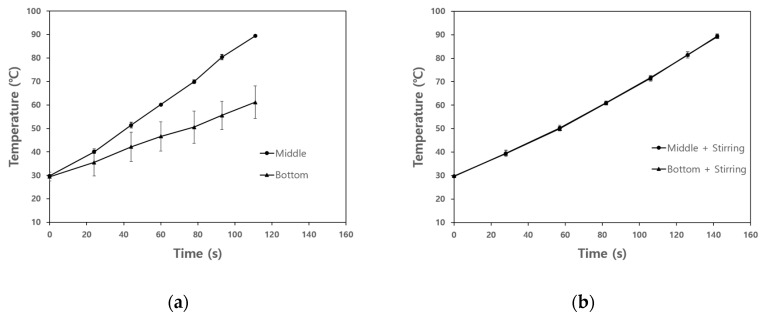
Temperature difference between middle and bottom of base solution: (**a**) without agitation, (**b**) with agitation.

Figure 6 shows the temperature difference of the base solution at different locations in the heating chamber during OH depending on the presence of agitation. The initial temperature of the base solution was approximately 30 °C regardless of location. When the agitation was not applied to OH, the temperature difference of the base solution at different locations significantly increased after 40 s (Figure 6a). The temperature at the middle of the heating chamber showed a rapid increase rate than the bottom. Although OH is known as the effective food thermal treatment to achieve temperature uniformity of single phase food, temperature non-uniformity was found in this study. 

As shown in Figure 6b, the temperature uniformity of the base solution regardless of location was achieved by agitation. The time required for the temperature of the middle or bottom to reach 90 °C was 144 s. The temperature difference between the middle and the bottom of the heating chamber was less than 1.4 °C during the entire heating process, and showed almost the same increase rate of temperature at all locations in the heating chamber. The forced convection by agitation was effective at achieving thermal uniformity of the base solution during OH. 

When the model food was treated by OH without agitation at atmospheric pressure, the temperatures of the base solution and solid particles were 90 ± 1.13 °C and 73.49 ± 0.38 °C, resulting in significant temperature difference. However, in the case of OH with agitation, the temperature difference between solid particles (90.2 ± 0.93 °C) and the base solution (87.29 ± 0.06 °C) was less than 3 °C. 

When multiphase food was treated by OH, the temperature difference between solid particles and liquid could be caused by limited convection. An excessive heat treatment is required to increase the temperature of solid particles; however, it will cause over-heating that deteriorates food quality [38]. In this study, it was found that forced convection by agitation was effective in resolving non-uniform temperature distribution in multiphase food.

The temperature values of the solid particles and base solution under OH–VC combination heating with agitation are summarized in Table 3. The temperature of solid particles was not significantly affected by the vacuum pretreatment time at constant vacuum pressure; however, the effect of vacuum pressure intensity was dominant in the temperature change of solid particles. The boiling point of the model food was lowered by the effect of OH–VC combination with agitation. The solid particles were only heated to the boiling point temperature of the base solution, and the final heating temperature of the base solution at vacuum gauge pressures of 0.8, 0.5, and 0.2 bar regardless of vacuum pretreatment time was 88.99 ± 0.42, 77.89 ± 0.69, and 57.45 ± 0.26 °C, respectively. The temperature difference between solid particles and the base solution under all conditions of OH–VC combination with agitation was within 3 °C. Since vacuum pretreatment expelled the air inside the solid and made the electrical conductivity of the solution and solid almost similar, it was possible to minimize the temperature difference between solid particles and base solution [39]. The OH–VC combination heating with agitation was effective at improving the temperature uniformity of multiphase food and preventing excessive thermal treatment.

### 3.4. Variation of Particle Hardness

The average hardness of the solid particles treated by OH with/without agitation was 215.7 ± 6.4 and 119.8 ± 10.1 N/m^2^, respectively. Since the solid particles in the model food were under-processed by OH without agitation, a relatively low hardness value was measured. 

**Figure 7:**
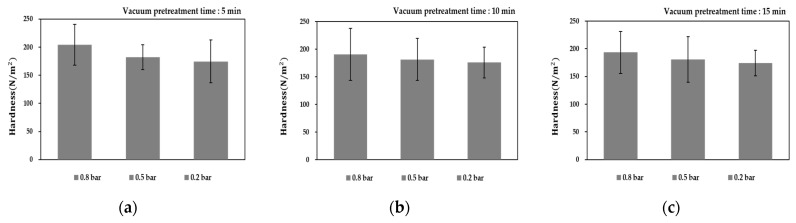
Change in hardness of solid particles under different OH–VC combination heating conditions with agitation: vacuum pretreatment time for (**a**) 5 min, (**b**) 10 min, and (**c**) 15 min at different vacuum intensity levels.

Figure 7 indicates the hardness change of solid particles in the model food treated by different OH–VC combination heating conditions with agitation. An increase in vacuum intensity had a great effect on the change in hardness of solid particles. This result is consistent with the previous studies showing that the firmness of papaya treated by vacuum the firmness was reduced compared to the atmospheric pressure treatment [40,41,42,43,44]. However, vacuum pretreatment time was not significant to change the hardness of solid particles. The lowest hardness of solid particles was observed at a vacuum gauge pressure of 0.2 bar and the hardness range was between 170 and 180 N/m^2^ under all pretreatment time conditions. Older people suffering from eating disorders prefer soft foods over tough or hard foods. Therefore, the texture of solid particles is a major factor to be considered in the processing of senior-friendly food consisting of solid and liquid phase food. The OH–VC combination heating made the texture of solids softer than individual OH treatment. In addition, this combination heating was suitable for the processing of the multiphase form of senior-friendly food.

### 3.5. Simulation Verification

#### 3.5.1. The Simulated Electric Field Strength Distribution in Ohmic Chamber 

**Figure 8:**
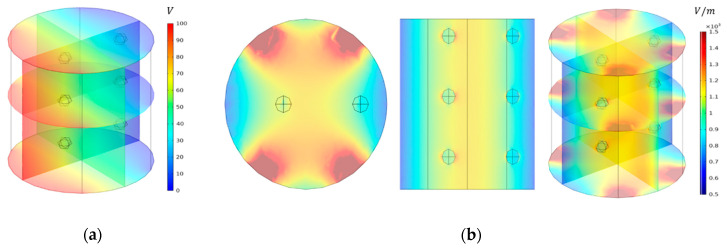
Simulated (**a**) electric potential and (**b**) electric field distributions.

The electric field distribution inside the OH chamber filled with model food was simulated using an AC/DC module in COMSOL Multiphysics (COMSOL 5.5, COMSOL, Inc., Palo Alto, CA, USA), as shown in Figure 8. The applied voltage to the simulation was 100 V. The electric field overshoot (approximately 3.4 kV/m) was determined at both edges of the curved rectangular electrodes. The current density was higher in the areas of both edges than in other parts. The electric field strength range in the center of the OH chamber was estimated between 1100 and 1200 V/m. In addition, the relatively low electric field strength range (740 to 870 V/m) was observed at the area between the edges of the electrodes. The electric field strength of the solution surrounding the solid particle was observed to be slightly higher than that of other parts, which seems to be a phenomenon that occurs when an electrical current passes through the material composed of different phases having different physical and electrical properties. Moreover, this phenomenon could result in the electric field interruption and non-uniform temperature distribution in the model food. 

#### 3.5.2. Temperature Distribution of Model Food under OH Depending on Presence of Agitation 

**Figure 9:**
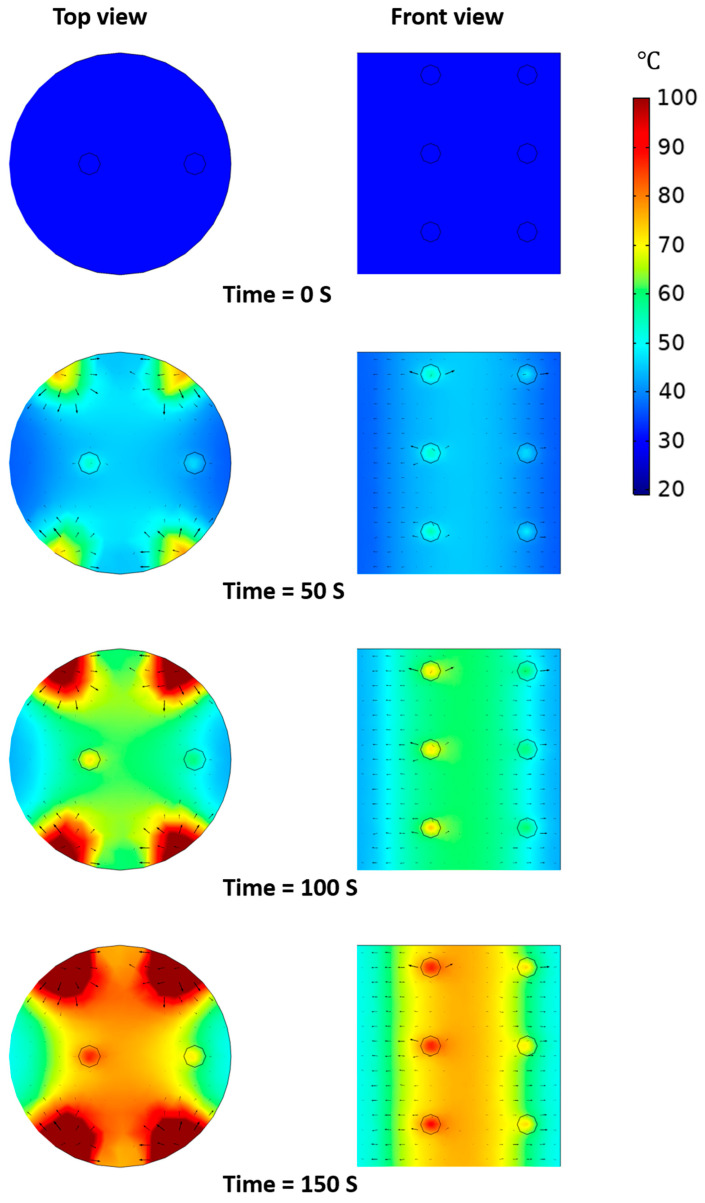
Simulated heating pattern of model food under OH without agitation.

Figure 9 shows the simulated heating pattern of model food under OH without agitation. The black arrows represent the total heat flux including conduction and convective heat flow. The rapid increase of temperature near both edges of the electrodes significantly affected the temperature distribution of model food inside the chamber. As the electric overshoot near both edges of the electrodes was estimated in the simulation of electric field distribution, it was predicted that the area near the edges of the electrodes was heated faster than other areas. In the top view of the simulation, the total heat flux was diffused from both edges of the electrodes. However, as shown in the front view, the total heat flux spreads outward from the center. Therefore, when the agitation was not applied to the OH of the model food, the generated internal heat from OH could not be uniformly distributed in the model food because of the limited natural convection. The temperature difference between the center and the outside was about 20 °C due to the non-uniform heating pattern. Even though the electrical conductivity of the solid particles was lower than that of the base solution, the heating rate of the solid particles was sharper than that of the base solution in the simulation. Since the locations of solid particles were close to the center of the chamber, the generated heat from OH affected the increase in temperature of the solid particles. The temperature difference between solid particles and base solution increased as heating time increased. The temperature range of the pork particles obtained from the experimental data of OH without agitation was from 60 to 81 °C. The simulation results showed that the temperature of the particle located in the center was about 83 °C and the outside was about 64 °C, which was very similar to the experimental data. The temperature difference between solid particles and base solution varied depending on the location of the solid particles and the difference range was from a minimum of 10 °C to a maximum of 30 °C.

**Figure 10:**
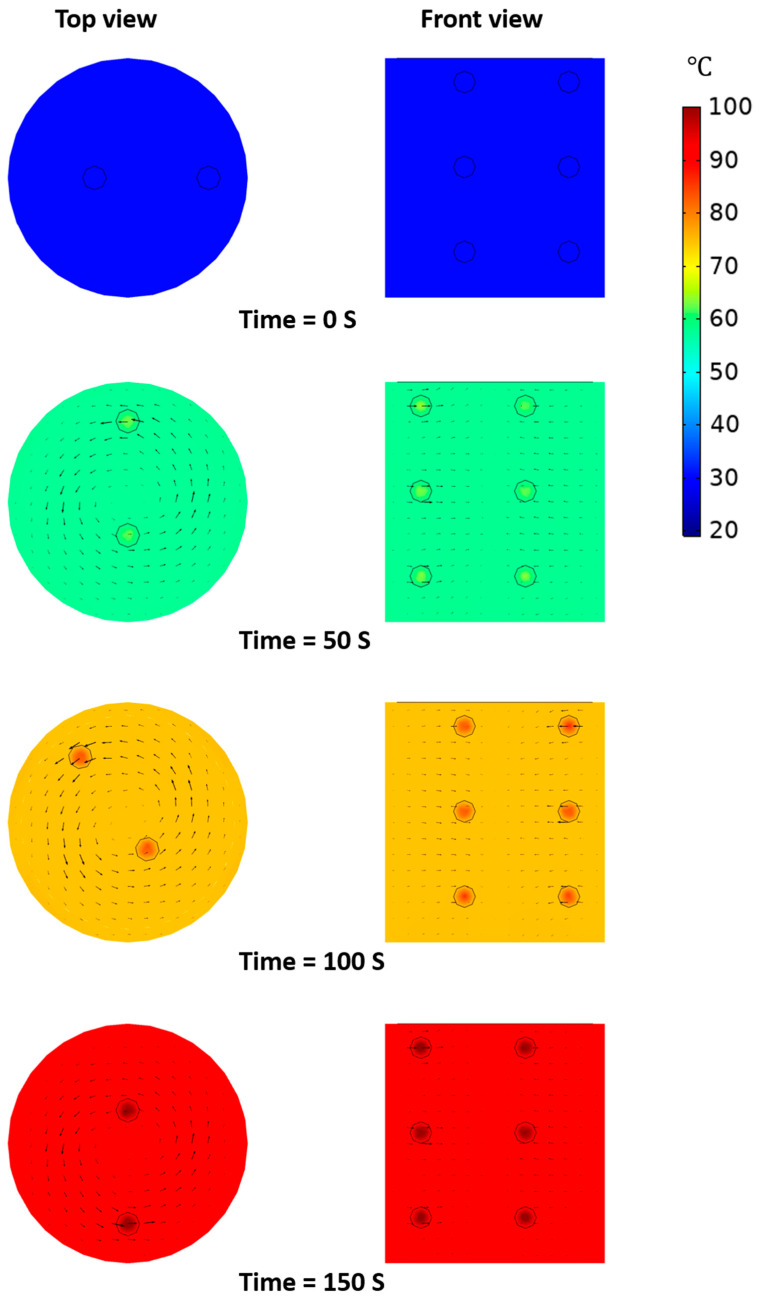
Simulated heating pattern of model food under OH with agitation.

Figure 10 shows the simulation results of the heating pattern of the model food under OH with agitation. The direction of the total heat flux was the same as the agitation direction. The exacerbated non-uniform temperature distribution between solid particles and base solution was minimized by the effect of forced convection. Regardless of the solid particle locations, solid particles were equally heated and had a similar heating rate. The temperature difference between solid particles and base solution was around 5 °C at all heating times and locations; however, the heating rates of solid particles and base solution showed a similar tendency. The simulated temperature values for solid particles and base solution were in good agreement with the experimental data and the maximum prediction error was about 3 °C. In this study, heating patterns of model food under OH with agitation could be effectively predicted through the simulation. 

## 4. Conclusions

The effects of vacuum and agitation on thermal uniformity of the model food under OH was evaluated in this study. By combining vacuum and agitation in OH of multiphase food, the boiling point of the base solution was lowered and thermal uniformity of model food was improved with softening of solid particles. In addition, the excessive heat treatment inside multiphase food was prevented. The simulation models for the OH of the model food with/without agitation were in good agreement with experimental data. In the simulation for the OH of the model food without agitation, solid particles were heated more rapidly than the base solution. The temperature difference between solid particles, depending on the locations in OH chamber (center and outside), increased with an increase in heating time. The simulation for the OH of the model food with agitation showed thermal uniformity between solid particles and base solution with a maximum difference within 5 °C. The developed OH–VC combination heating with agitation has great potential to process senior-friendly foods with improvement of the texture of solid phase food and enhanced thermal uniformity. 

## Figures and Tables

**Table 1:** Electrical conductivities of commercialized senior-friendly foods and base solution at different temperatures.

Temperature (°C)	A	B	C	D	E	F	Base Solution
30	0.76 ± 0.07	1.11 ± 0.01	0.92 ± 0.03	0.39 ± 0.01	0.27 ± 0.01	0.75 ± 0.04	0.79 ± 0.03
40	0.91 ± 0.05	1.32 ± 0.01	1.10 ± 0.05	0.45 ± 0.02	0.31 ± 0.01	0.93 ± 0.06	0.90 ± 0.02
50	1.08 ± 0.07	1.56 ± 0.02	1.23 ± 0.04	0.53 ± 0.02	0.37 ± 0.01	1.12 ± 0.07	1.01 ± 0.02
60	1.20 ± 0.06	1.82 ± 0.04	1.37 ± 0.08	0.60 ± 0.02	0.43 ± 0.01	1.33 ± 0.08	1.12 ± 0.03
70	1.35 ± 0.08	2.00 ± 0.08	1.62 ± 0.11	0.69 ± 0.02	0.47 ± 0.01	1.53 ± 0.09	1.22 ± 0.03
80	1.48 ± 0.06	2.29 ± 0.07	1.76 ± 0.05	0.75 ± 0.04	0.53 ± 0.02	1.67 ± 0.11	1.31 ± 0.04
90	1.58 ± 0.04	2.39 ± 0.07	1.85 ± 0.09	0.82 ± 0.04	0.60 ± 0.01	1.86 ± 0.10	1.45 ± 0.06

Types of commercialized senior-friendly foods: soft diet (A and B), liquid food (C, D, and E), and pudding (F).

**Table 2:** The measured electrical conductivities of pork particle and base solution after vacuum pretreatment.

			30 °C	40 °C	50 °C	60 °C	70 °C	80 °C	90 °C
Base solution (A)			0.80 ± 0.03	0.90 ± 0.02	1.01 ± 0.02	1.13 ± 0.03	1.22 ± 0.03	1.31 ± 0.04	1.45 ± 0.06
Pork particle (B)			0.74 ± 0.03	0.83 ± 0.04	0.95 ± 0.06	1.03 ± 0.05	1.12 ± 0.06	1.24 ± 0.06	1.35 ± 0.07
1 bar	5 min	(A)	0.86 ± 0.02	0.96 ± 0.03	1.06 ± 0.03	1.16 ± 0.01	1.28 ± 0.03	1.39 ± 0.01	1.54 ± 0.01
		(B)	0.79 ± 0.79	0.90 ± 0.09	1.00 ± 0.10	1.10 ± 0.10	1.19 ± 0.10	1.28 ± 0.11	1.38 ± 0.11
	10 min	(A)	0.86 ± 0.01	0.95 ± 0.01	1.05 ± 0.01	1.17 ± 0.02	1.29 ± 0.02	1.39 ± 0.04	1.47 ± 0.02
		(B)	0.72 ± 0.05	0.88 ± 0.04	0.98 ± 0.04	1.07 ± 0.05	1.17 ± 0.05	1.27 ± 0.07	1.38 ± 0.06
	15 min	(A)	0.86 ± 0.04	0.95 ± 0.03	1.06 ± 0.03	1.18 ± 0.03	1.30 ± 0.06	1.39 ± 0.05	1.48 ± 0.04
		(B)	0.78 ± 0.05	0.94 ± 0.08	1.06 ± 0.09	1.14 ± 0.08	1.23 ± 0.09	1.32 ± 0.09	1.43 ± 0.10
0.8 bar	5 min	(A)	0.82 ± 0.04	0.92 ± 0.02	1.04 ± 0.04	1.15 ± 0.02	1.26 ± 0.05	1.37 ± 0.05	1.49 ± 0.06
		(B)	0.79 ± 0.09	0.99 ± 0.12	1.15 ± 0.14	1.24 ± 0.15	1.32 ± 0.13	1.43 ± 0.12	1.51 ± 0.11
	10 min	(A)	0.84 ± 0.06	0.93 ± 0.05	1.03 ± 0.04	1.14 ± 0.03	1.26 ± 0.03	1.37 ± 0.04	1.48 ± 0.05
		(B)	0.75 ± 0.09	0.89 ± 0.10	1.02 ± 0.07	1.13 ± 0.07	1.22 ± 0.09	1.33 ± 0.08	1.44 ± 0.09
	15 min	(A)	0.84 ± 0.02	0.94 ± 0.02	1.04 ± 0.02	1.15 ± 0.02	1.26 ± 0.03	1.36 ± 0.03	1.46 ± 0.01
		(B)	0.75 ± 0.06	0.88 ± 0.05	1.00 ± 0.04	1.09 ± 0.05	1.20 ± 0.03	1.31 ± 0.03	1.41 ± 0.03
0.5 bar	5 min	(A)	0.84 ± 0.02	0.95 ± 0.02	1.06 ± 0.02	1.17 ± 0.03	1.29 ± 0.05	1.40 ± 0.08	1.48 ± 0.07
		(B)	0.72 ± 0.07	0.83 ± 0.05	0.97 ± 0.04	1.09 ± 0.03	1.23 ± 0.07	1.35 ± 0.08	1.47 ± 0.07
	10 min	(A)	0.81 ± 0.05	0.91 ± 0.04	1.02 ± 0.04	1.13 ± 0.04	1.25 ± 0.03	1.37 ± 0.02	1.47 ± 0.03
		(B)	0.70 ± 0.06	0.81 ± 0.06	0.93 ± 0.06	1.04 ± 0.04	1.16 ± 0.05	1.25 ± 0.05	1.35 ± 0.06
	15 min	(A)	0.84 ± 0.02	0.94 ± 0.02	1.05 ± 0.03	1.16 ± 0.03	1.28 ± 0.03	1.38 ± 0.03	1.48 ± 0.04
		(B)	0.74 ± 0.04	0.85 ± 0.03	0.96 ± 0.04	1.05 ± 0.03	1.14 ± 0.03	1.23 ± 0.03	1.33 ± 0.04
0.2 bar	5 min	(A)	0.84 ± 0.04	0.98 ± 0.08	1.10 ± 0.10	1.22 ± 0.11	1.31 ± 0.12	1.43 ± 0.10	1.51 ± 0.06
		(B)	0.75 ± 0.04	0.86 ± 0.06	0.98 ± 0.06	1.08 ± 0.05	1.18 ± 0.04	1.28 ± 0.04	1.39 ± 0.04
	10 min	(A)	0.87 ± 0.04	0.98 ± 0.05	1.09 ± 0.07	1.22 ± 0.07	1.35 ± 0.08	1.48 ± 0.09	1.55 ± 0.05
		(B)	0.72 ± 0.08	0.85 ± 0.07	0.98 ± 0.07	1.10 ± 0.09	1.23 ± 0.10	1.35 ± 0.11	1.44 ± 0.08
	15 min	(A)	0.82 ± 0.06	0.93 ± 0.03	1.03 ± 0.02	1.13 ± 0.02	1.25 ± 0.05	1.38 ± 0.07	1.47 ± 0.04
		(B)	0.74 ± 0.06	0.85 ± 0.06	0.97 ± 0.07	1.07 ± 0.07	1.15 ± 0.07	1.24 ± 0.09	1.36 ± 0.11

**Table 3:** Temperature values of model food under different ohmic heating–vacuum (OH–VC) combination heating with agitation.

			Vacuum Pretreatment Time			
			0 min	5 min	10 min	15 min
Vacuum intensity	0.8 bar	(A)	88.99 ± 0.42	88.65 ± 0.15	89.14 ± 0.72	87.77 ± 0.13
		(B)	88.19 ± 2.26	86.68 ± 1.15	86.75 ± 1.27	86.09 ± 0.37
	0.5 bar	(A)	77.89 ± 0.69	77.12 ± 0.66	76.62 ± 0.34	76.95 ± 0.08
		(B)	78.23 ± 1.58	75.97 ± 0.50	76.18 ± 0.21	74.81 ± 2.75
	0.2 bar	(A)	54.93 ± 0.69	54.59 ± 0.19	54.4 ± 0.25	54.43 ± 0.50
		(B)	54.18 ± 1.93	53.82 ± 0.04	53.85 ± 1.51	53.81 ± 1.76

(A) is the base solution, (B) is solid particles.

## Data Availability

Data presented in this study are available in the article.

## References

[1] Bloom D.E., Canning D., Lubet A. (2015). Global Population Aging: Fact, Challenges, Solutions & Perspectives. Chall. Solut. Perspect. Daedalus.

[2] Suzuki T. (2013). Low Fertility and Population Aging in Germany and Japan: Prospects and Policies. Fertility and Public Policy.

[3] Maestas N., Mullen K.J., Powell D. (2016). The Effect of Population Aging on Economic Growth, the Labor Force and Productivity (No. w22452).

[4] Baek J. (2017). Trends in elderly-friendly home-style alternative food. Inst. Electron. Inf. Eng..

[5] Lee S.B. (2017). *Trend of Japan’s Care Food Industry*; Seoul, Korea. https://www.kati.net/board/pubilshedMaterialsView.do?menu_dept2=48&board_seq=85502.

[6] Hsu M., Yamada T. (2019). Population Aging, Health Care, and Fiscal Policy Reform: The Challenges for Japan. Scand. J. Econ..

[7] García J., Méndez D., Álvarez M., Sanmartin B., Vazquez Sobrado R., Regueiro L., Atanassova M. (2019). Design of novel functional food products enriched with bioactive extracts from holothurians for meeting the nutritional needs of the elderly. LWT Food Sci. Technol..

[8] Eggersdorfer M., Akobundu U., Bailey R.L., Shlisky J., Beaudreault A.R., Bergeron G., Blancato R.B., Blumberg J.B., Bourassa M.W., Gomes F. (2018). Hidden hunger: Solutions for America’s aging populations. Nutrients.

[9] Bailey R.L., Harris Ledikwe J., Smiciklas-Wright H., Mitchell D.C., Jensen G.L. (2004). Persistent oral health problems associated with comorbidity and impaired diet quality in older adults. J. Am. Diet. Assoc..

[10] Ahmed T., Haboubi N. (2010). Assessment and management of nutrition in older people and its importance to health. Clin. Interv. Aging.

[11] Deutz N.E.P., Bauer J.M., Barazzoni R., Biolo G., Boirie Y., Bosy-Westphal A., Cederholm T., Cruz-Jentoft A., Krznariç Z., Nair K.S. (2014). Protein intake and exercise for optimal muscle function with aging: Recommendations from the ESPEN Expert Group. Clin. Nutr..

[12] Schiffman S.S., Graham B.G. (2000). Taste and smell perception affect appetite and immunity in the elderly. Eur. J. Clin. Nutr..

[13] Scharfstein M. (2006). Gaurf Food Industry for The Aging Society. Food Sci. Ind..

[14] Torkian Boldaji M., Borghei A.M., Beheshti B., Hosseini S.E. (2015). The process of producing tomato paste by ohmic heating method. J. Food Sci. Technol..

[15] Hashemi S.M.B., Gholamhosseinpour A., Niakousari M. (2019). Application of microwave and ohmic heating for pasteurization of cantaloupe juice: Microbial inactivation and chemical properties. J. Sci. Food Agric..

[16] Fadavi A., Yousefi S., Darvishi H., Mirsaeedghazi H. (2018). Comparative study of ohmic vacuum, ohmic, and conventional-vacuum heating methods on the quality of tomato concentrate. Innov. Food Sci. Emerg. Technol..

[17] Nguyen L.T., Choi W., Lee S.H., Jun S. (2013). Exploring the heating patterns of multiphase foods in a continuous flow, simultaneous microwave and ohmic combination heater. J. Food Eng..

[18] Jun S., Sastry S. (2005). Modeling and optimization of ohmic heating of foods inside a flexible package. J. Food Process Eng..

[19] Sastry S.K., Palaniappan S. (1992). Mathematical Modeling and Experimental Studies on Ohmic Heating of Liquid-Particle Mixtures in a Static Heater. J. Food Process Eng..

[20] Benabderrahmane Y., Pain J.P. (2000). Thermal behaviour of a solid/liquid mixture in an ohmic heating sterilizer-slip phase model. Chem. Eng. Sci..

[21] Fadavi A., Salari S. (2019). Ohmic Heating of Lemon and Grapefruit Juices Under Vacuum Pressure—Comparison of Electrical Conductivity and Heating Rate. J. Food Sci..

[22] Wang L., Sun D.-W. (2002). Modelling vacuum cooling process of cooked meat—part 1: Analysis of vacuum cooling system Mode ` me de pour la viande cuite—Partie 1: Analyse du syste refroidissement sous vide. Int. J. Refrig..

[23] Yaghmaee P., Durance T.D. (2005). Destruction and injury of Escherichia coli during microwave heating under vacuum. J. Appl. Microbiol..

[24] Paranjpe S.S., Ferruzzi M., Morgan M.T. (2012). Effect of a flash vacuum expansion process on grape juice yield and quality. LWT Food Sci. Technol..

[25] Mariscal M., Bouchon P. (2008). Comparison between atmospheric and vacuum frying of apple slices. Food Chem..

[26] Darvishi H., Mohammadi P., Fadavi A., Koushesh Saba M., Behroozi-Khazaei N. (2019). Quality preservation of orange concentrate by using hybrid ohmic—Vacuum heating. Food Chem..

[27] Choi W., Kim S.S., Park S.H., Ahn J.B., Kang D.H. (2020). Numerical analysis of rectangular type batch ohmic heater to identify the cold point. Food Sci. Nutr..

[28] Ye X., Ruan R., Chen P., Doona C. (2004). Simulation and verification of ohmic heating in static heater using MRI temperature mapping. LWT Food Sci. Technol..

[29] Zhu S.M., Zareifard M.R., Chen C.R., Marcotte M., Grabowski S. (2010). Electrical conductivity of particle-fluid mixtures in ohmic heating: Measurement and simulation. Food Res. Int..

[30] Chen C., Abdelrahim K., Beckerich I. (2010). Sensitivity analysis of continuous ohmic heating process for multiphase foods. J. Food Eng..

[31] Marra F., Zell M., Lyng J.G., Morgan D.J., Cronin D.A. (2009). Analysis of heat transfer during ohmic processing of a solid food. J. Food Eng..

[32] Hashemi S.M.B., Roohi R. (2019). Ohmic heating of blended citrus juice: Numerical modeling of process and bacterial inactivation kinetics. Innov. Food Sci. Emerg. Technol..

[33] Salengke S., Sastry S.K. (2007). Models for ohmic heating of solid-liquid mixtures under worst-case heating scenarios. J. Food Eng..

[34] Zell M., Cronin D.A., Morgan D.J., Marra F., Lyng J.G. Solid Food Pasteurization by Ohmic Heating: Influence of Process Parameters. Proceedings of the COMSOL Conference.

[35] Jun S., Sastry S. (2007). Reusable pouch development for long term space missions: A 3D ohmic model for verification of sterilization efficacy. J. Food Eng..

[36] Wilcox D.C. (1998). Turbulence Modeling for CFD.

[37] Wang W.C., Sastry S.K. (1993). Salt diffusion into vegetable tissue as a pretreatment for ohmic heating: Electrical conductivity profiles and vacuum infusion studies. J. Food Eng..

[38] Shim J., Lee S.H., Jun S. (2010). Modeling of ohmic heating patterns of multiphase food products using computational fluid dynamics codes. J. Food Eng..

[39] Allali H., Marchal L., Vorobiev E. (2010). Effects of vacuum impregnation and ohmic heating with citric acid on the behaviour of osmotic dehydration and structural changes of apple fruit. Biosyst. Eng..

[40] Moreno J., Bugueño G., Velasco V., Petzold G., Tabilo-Munizaga G. (2004). Osmotic dehydration and vacuum impregnation on physicochemical properties of Chilean papaya (Carica candamarcensis). J. Food Sci..

[41] Moreno J., Simpson R., Estrada D., Lorenzen S., Moraga D., Almonacid S. (2011). Effect of pulsed-vacuum and ohmic heating on the osmodehydration kinetics, physical properties and microstructure of apples (cv. Granny Smith). Innov. Food Sci. Emerg. Technol..

[42] Moreno J., Simpson R., Baeza A., Morales J., Muñoz C., Sastry S., Almonacid S. (2012). Effect of ohmic heating and vacuum impregnation on the osmodehydration kinetics and microstructure of strawberries (cv. Camarosa). LWT Food Sci. Technol..

[43] Moreno J., Zúñiga P., Dorvil F., Petzold G., Mella K., Bugueño G. (2017). Osmodehydration assisted by ohmic heating/pulse vacuum in apples (cv. Fuji): Retention of polyphenols during refrigerated storage. Int. J. Food Sci. Technol..

[44] Iborra-Bernad C., Tárrega A., García-Segovia P., Martínez-Monzó J. (2014). Comparison of Vacuum Treatments and Traditional Cooking Using Instrumental and Sensory Analysis. Food Anal. Methods.

